# Associations of combined exposure to selected metal mixtures with thyroid hormones in children: a cross-sectional study in China

**DOI:** 10.3389/fpubh.2025.1387702

**Published:** 2025-01-22

**Authors:** Yuhan Cao, Shiting Xiang, Yuwei Du, Meiling Chen, Rumeng Xue, Qi Li, Jun Qiu, Yanying Duan

**Affiliations:** ^1^Department of Occupational and Environmental Health, Xiangya School of Public Health, Central South University, Changsha, China; ^2^Hunan Children's Research Institute (HCRI), The Affiliated Children's Hospital of Xiangya School of Medicine, Central South University (Hunan Children's Hospital), Changsha, China; ^3^Hunan Institute for Drug Control, Changsha, China

**Keywords:** metals, thyroid hormones, children, mixture exposure, quantile-based g-computation (QGC)

## Abstract

**Background:**

Exposure to several metal elements has been found to be associated with thyroid hormone homeostasis. However, evidence for combined exposure is inconclusive, especially for children.

**Objective:**

To examine the individual and joint effects of blood metal elements on thyroid hormones in children.

**Methods:**

A total of 12,470 children aged 0–14 were collected from January 2018 to December 2021 in Hunan Children's Hospital. The concentrations of lead (Pb), iron (Fe), calcium (Ca), copper (Cu), zinc (Zn) and magnesium (Mg) in blood were detected via atomic absorption spectrometry (AAS). The levels of thyroid stimulating hormone (TSH), triiodothyronine (TT3, FT3) and total and free thyroxine (TT4, FT4) were measured by electrochemiluminescence immunoassay (ECLIA). Generalized linear regression (GLR) model and Quantile-based g-computation (QGC) were employed to estimate the association between metal exposure and thyroid hormone homeostasis.

**Results:**

GLR model showed that a unit increase in ln-transformed Fe was associated with increases in TT3 (β = 0.163; *P*_FDR_ < 0.001), TT4 (β = 12.255; *P*_FDR_ < 0.001) and FT3 (β = 0.615; *P*_FDR_ < 0.001), as well as decreases in TSH (β = −0.471; *P*_FDR_ = 0.005) and FT4 (β = −1.938; *P*_FDR_ < 0.001). The result of QGC analysis indicated a positive relationship of the ln-transformed concentration of metal mixture with the levels of TT3 (β = 0.018; *P* = 0.012), TT4 (β = 2.251; *P* < 0.001) and FT3 (β = 0.074; *P* < 0.001) in children. Fe was the predominant contributor among the metal mixture with positive contributions to TT3 (weight = 0.439), TT4 (weight = 0.502) and FT3 (weight = 0.450).

**Conclusions:**

The combined metal exposure was associated with increased levels of TT3, TT4, and FT3 in children and Fe appeared to be the major contributor. Further studies are warranted to confirm our findings and elucidate the underlying mechanisms.

## 1 Introduction

Thyroid gland is the largest endocrine gland in human body, responsible for synthesis, storage and secretion of thyroid hormones (THs), such as Triiodothyronine (T3) and Thyroxine (T4) (1). T3 is the metabolically active form of THs while T4 is considered a precursor or prohormone, which are both tightly regulated through feedback inhibition by thyroid stimulating hormone (TSH) (2). THs are excitatory hormones that maintain cell growth and body metabolism, as well as mediate the development and differentiation of cardiovascular system, central nervous system and reproductive system (3, 4). Disrupted thyroid homeostasis has been associated with altered neural differentiation and cognitive deficits, as well as metabolic disorders (5–7).

In recent years, there has been increasing interest in the potential influence of element on the homeostasis of THs (8–10). Due to the unique property, lead (Pb) was used to make drinking water pipes and lead-acid battery, as well as added into paint (11, 12). Children in China were widely exposed to lead through drinking water and environmental sources with blood Pb level still higher than that in developed countries (13). Experimental studies shown that Pb could disturb endocrine function of thyroid through decreasing thyroid hormone secretion and endocrine-related gene expression as well as changing the tissue structure (14, 15). Iron (Fe), magnesium (Mg), copper (Cu), zinc (Zn) and calcium (Ca) are belong to essential element. Deficiency in essential element is the most common nutritional disorder detrimental to the neural, behavior and cognitive performance development of children (16) and probably alter thyroid hormone levels in experimental animals (17–20). Epidemiological studies regarding the effect of element on thyroid hormone homeostasis focused more on adults (21–23), and the results were quite different from those of children (24). Among children, the limited investigation did not reach consistent conclusions. Bucci et al. (25) found that Zn supplementation could reduce TSH level in hypozincemic Down children. Nevertheless, another investigation by Marreiro et al. (26) reported that intervention with Zn showed no influence on the metabolism of thyroid hormones among children with Down syndrome. Blood and hair Pb levels were positively correlated with TSH level while negatively associated with free thyroxine (FT4) level in children living in a rural community of Southern Brazil (27). Conversely, a recent study conducted in China showed that blood Pb level was inversely associated with the serum TSH level among school-age children living nearby a Pb-Zn mine (28).

Alterations in THs homeostasis during infancy, childhood and puberty, even subtle changes, may lead to growth delay, neuropsychological development delay and precocious puberty (29, 30). Therefore, there is a pressing need for research to explore the relationship between metal elements and thyroid hormones, especially in children (31). Notably, human body is simultaneously exposed to multiple metals, which result in a combined health effect quite different from the effect of single element exposure. However, few studies evaluated the joint effect of metal mixture exposure on THs in children (32).

In the present research, the physical examination data of 12,470 children aged 0–14 years in Hunan Children's Hospital from 2018 to 2021 were retrospectively analyzed. The impact of single selected metal (i.e., Pb, Fe, Ca, Cu, Zn, and Mg) on THs was analyzed by generalized linear model (GLM) and restricted cubic splines (RCS). Furthermore, quantile-based g-computation (QGC) model was applied to estimate the effect of metal co-exposure on THs and identify the most important individual metal in the mixture.

## 2 Materials and methods

### 2.1 Participants

This cross-sectional study was conducted in the Department of Children Health Care, Hunan Children's Hospital in Changsha city, China. Children who received medical examinations at outpatient clinic between 1st January 2018 and 31st December 2021 were recruited in this study based on the inclusion criteria: (1) between 0 and 14 years old, (2) they and their parents were residents of Hunan Province. Children's anthropometric data (height, weight) and blood samples were collected. Medical history and other basic information were collected by interviewing parents or guardians. Children with thyroid diseases (such as hyperthyroidism and hypothyroidism) and/or treated with any thyroid interfering drugs were excluded. At last, a total of 12,470 children were enrolled in this study. The flow chart of participant selection was shown in Supplementary Figure S1. All the processes were approved by the Ethics Committee of Hunan Children's Hospital.

### 2.2 Collection of blood sample and determination of element concentration

About 2 mL of fasting venous blood was collected from each participant in the Department of Children Health Care by trained nurse, and then analyzed in the clinical laboratory of Hunan Children's Hospital. Before metal concentration measurement, standard curves of these selected metals were conducted by serial dilution of the stock standard solutions. Sample digestion was prepared by mixing 0.5 mL of blood sample with 4.5 mL of 0.5% Triton X-100 solution containing 1% HNO3 in a tube. Then after homogenization, the samples were detected immediately by a 7030A atomic absorption spectrometer (East-West Analytical Instruments Co., Beijing, China) for the concentrations of Fe, Ca, Cu, Zn and Mg. The content of Pb was determined by a Z-2700 atomic absorption spectrometer (Hitachi, Tokyo, Japan) through graphite furnace atomic absorption spectrometry. Coefficients of variation in both intra- and inter-assay were < 5.0%. Each sample was analyzed twice, and the average value was taken as the final value. ClinChek controls levels 1 and 2 (Recipe Chemicals, Munich, Germany) were used as quality control materials and the recovery rates were between 82% and 106%. Reference value ranges for the whole blood element concentrations in our laboratory were as follows: Fe: 363~474 μg/mL, Ca: 56~78.8 μg/mL, Cu: 0.75~2.50 μg/mL, Zn: 4.8~9.3 μg/mL, Mg: 27.1~45.5 μg/mL, Pb: 0~50 μg/L.

### 2.3 Estimation of thyroid hormones levels

Serum was separated from whole blood sample by centrifugation at 1,000 rpm, 10 min and stored at −20°C until analyses. Plasma concentrations of total and free T3 and T4 (TT3, FT3, TT4, and FT4) were all measured by using an automated electrochemiluminescence immunoassay (ECLIA) on the Roche Cobas e 411 analyzer as well as manufacturer-recommended reagents and calibrators (Roche Diagnostics GmbH, Mannheim, Germany). The TSH level was measured by using a 1-step sandwich method of ECLIA, according to the producer's instructions. The internal quality control serum samples were randomly measured along with the study samples to ensure the accuracy of the analytical methods. The analytical sensitivities of TSH, TT3, TT4, FT3 and FT4 were 0.27 μIU/mL, 0.3 nmol/L, 5.4 nmol/L, 0.4 pmol/L and 0.3 pmol/L, respectively.

### 2.4 Covariates

Potential confounding factors were adjusted in the analysis, including children's age (years), gender (girl/boy), parent smoking (yes/no), parent history of thyroid disease (yes/no), maternal education level (middle school or below/high school/college or above), urinary iodine concentration (μg/L), season (spring/summer/fall/winter) and BMI (kg/m^2^). Smoking was defined as smoking at least one cigarette a day for the past year. Thyroid disease included hyperthyroidism, hypothyroidism, thyroiditis, goiter and thyroid cancer. BMI was calculated as weight divided by squared height. Thyroid hormone levels fluctuated with season (33, 34), and therefore, the results of THs were classified into four groups based on the season for blood samples collection (spring: 1 March-31 May; summer: 1 June-31 August; fall: 1 September-30 November; winter: 1 December-28 or 29 February).

### 2.5 Statistical analysis

We calculated summary statistics to describe the basic characteristics for participants, FHs levels and the distribution of metal. For demographics, continuous variables were expressed as mean ± standard deviation (SD) or median (P25, P75), and categorical variables were presented as counts and percentages. Because of the skewness distribution of the metal concentrations, they were ln-transformed to make them normally distributed. Bivariate correlations between metallic element concentrations were explored by Spearman's rank test (continuous variables).

Generalized linear regression (GLR) model was used to calculate the regression coefficient (β) and 95% confidence intervals (CIs) for the associations of serum THs concentrations with both single metal and multiple metals. Metallic element and thyroid hormone were treated as continuous variables in the model and all the analyses were adjusted for sex, age, BMI and season. We used the Benjamini–Hochberg false discovery rate (FDR) to adjust all *P* values. Then, restricted cubic spline (RCS) was used to investigate the potential dose-response relationships between individual metal and thyroid hormones. The ln-transformed metal levels were treated as the independent variables and the thyroid hormone concentrations as the dependent variable. At last, QGC model was used to evaluate the association of metallic elements as a joint mixture with thyroid hormones, which yielded estimates of the effect for the increase in all the element exposures by one quantile. This analysis method, combining weighted quantile sum (WQS) regression and g-computation, relaxes the assumption that all chemical exposures are associated with the outcome in the same direction (35). Additionally, QGC model generally allows for flexible polynomial functions of the mixture exposure-response, though for simplicity we describe the implementation under linearity assumptions. The weight for each metal is calculated without bootstrapping, which represents the proportion of the contribution of a specific metal to thyroid hormone among the metals in the same direction, thus sum to 1 for positive and to −1 for negative direction, respectively. After adjusted for covariates, we investigated two different mixtures, i.e., a mixture containing 6 metallic elements and a mixture only containing 5 essential metal elements (Fe, Ca, Cu, Zn, and Mg). The overall mixture effect was interpreted as the effect on the outcome of increasing every ln-transformed exposure by one quantile (*q* = 4), the bootstrap variance with B was set as 500.

We performed three sensitivity analyses to test the reliability of our results. Firstly, considering that the QGC model may lead to counteracting effect among mixture (36), we also applied WQS to analyze the combined effects of metals exposure on thyroid hormones. Secondly, gender may modify the associations between metal elements and health outcomes (37, 38), we conducted stratified analyses among boys and girls, respectively, in the multiple-metal models. Similarly, we performed stratified analyzes separately for the four seasons in the multi-metal models.

GLR model was performed by using the Statistic Package for Social Sciences (SPSS), (v20.0; SPSS Inc., Chicago, IL, USA). We applied RCS, QGC and WQS analyses by R statistical software (v4.1.1; R Core Team 2021) by using R packages “rms,” “qgcomp,” and “gWQS,” respectively. In all statistical analyses, the criterion of significance was defined as *P* < 0.05.

## 3 Results

### 3.1 Characteristics of the study population and the distribution of metallic element

The demographic characteristics and the concentrations of THs among children were displayed in Table 1. A total of 12,470 children were recruited in this study with a mean age of 7.21 ± 3.64 years old, and the mean BMI was 15.69 ± 2.23 kg/m^2^. More than half of the participants were boys (61.8%) and the largest number of measurements were performed in summer (40.0%). The median levels for TSH, TT3, TT4, FT3, and FT4 were 2.57 uIU/mL, 2.30 nmol/L, 110.00 nmol/L,6.41 pmol/L and 15.93 pmol/L, respectively.

**Table 1:** Demographic characteristics and thyroid hormones levels of the study population (*n* = 12,470).

**Characteristics**	**Mean ±S.D or *N*(%) or median (P25, P75)**
Age (years)	7.21 ± 3.64
**Gender**	
Boy	7,712 (61.8)
Girl	4,758 (38.2)
Height (m)	1.12 ± 0.22
Weight (kg)	21.23 ± 9.70
BMI (kg/m^2^)	15.69 ± 2.23
**Parent smoking**	
Yes	7,461 (59.8)
No	5,009 (41.2)
**Parent history of thyroid disease**	
Yes	568 (4.6)
No	11,902 (95.4)
**Maternal education level**	
Middle school or below	325 (2.6)
High school	1,745 (14.0)
College or above	10,400 (83.4)
Urinary iodine concentration (μg/L)	181.5 (108, 303)
**Season**	
Spring	3,222 (25.8)
Summer	4,991 (40.0)
Fall	1,983 (15.9)
Winter	2,274 (18.2)
**Thyroid hormone profile**	
TSH (uIU/mL)	2.57 (1.83, 3.57)
TT3 (nmol/L)	2.30 (2.04, 2.59)
TT4 (nmol/L)	110.00 (97.69, 123.32)
FT3 (pmol/L)	6.41 (5.89, 6.98)
FT4 (pmol/L)	15.93 (13.11, 17.88)

Thyroid disease included hyperthyroidism, hypothyroidism, thyroiditis, goiter and thyroid cancer.

Table 2 demonstrated the distributions of metallic elements for the subjects. The median concentrations of Pb, Fe, Ca, Cu, Zn and Mg were 32.71 μg/L, 399.96 μg/mL, 63.47 μg/mL, 0.91 μg/mL, 6.39 μg/mL, and 35.94 μg/mL, respectively. Except for Cu and Fe, Cu and Ca, and Pb and Zn, other metals showed significantly weak correlations between each other (all *P* value < 0.05, the absolute value of *r* < 0.3, Supplementary Figure S2).

**Table 2:** Distribution of the metal concentrations among participants (*n* = 12,470).

**Metals**	**mean**	**5th**	**25th**	**50th**	**75th**	**95th**
Pb (μg/L)	34.40	13.18	24.54	32.71	42.20	60.09
Fe (μg/mL)	400.37	327.39	369.01	399.96	430.54	475.30
Ca (μg/mL)	63.93	53.24	58.46	63.47	68.87	77.10
Cu (μg/mL)	0.91	0.72	0.83	0.91	1.00	1.10
Zn (μg/mL)	6.44	4.69	5.54	6.39	7.25	8.57
Mg (μg/mL)	35.94	29.30	33.09	35.94	38.65	42.70

### 3.2 Associations between blood metals and serum thyroid hormones in single-metal and multiple-metal models

Table 3 showed the results of GLR model assessing the relationship between blood metals and serum thyroid hormones. In multiple-metal model, an ln-unit increase in Pb level was associated with higher TSH (β = 0.096; *P*_FDR_ = 0.013) and FT4 (β = 0.227; *P*_FDR_ < 0.001) after adjustment for the covariates. The concentration of Fe was positively associated with TT3 (β = 0.163; *P*_FDR_ < 0.001), TT4 (β = 12.255; *P*_FDR_ < 0.001) and FT3 (β = 0.615; *P*_FDR_ < 0.001) while negatively associated with TSH (β = −0.471; *P*_FDR_ = 0.005) and FT4 (β = −1.938; *P*_FDR_ < 0.001). There was a positive association between ln-Cu level and TT4 concentration (β = 8.403; *P*_FDR_ < 0.001). The concentration of ln-Mg was positively associated with TT3 (β = 0.079; *P*_FDR_ = 0.044) and TT4 (β = 5.467; *P*_FDR_ = 0.003). In addition, ln-Zn was negatively associated with TT3 (β = −0.083; *P*_FDR_ < 0.001) and FT3 (β = −0.169; *P*_FDR_ < 0.001) while positively associated with FT4 (β = 0.494; *P*_FDR_ = 0.003). And all the results in multiple-metal models were basically in line with the results in single-metal models.

**Table 3:** Associations of the ln-transformed blood metal levels with serum thyroid hormones analyzed by the generalized linear regression.

	**Single-metal model**		**Multiple-metal model**	
	β **(95% CI)**	*P*_FDR_	β **(95% CI)**	*P*_FDR_
**TSH**				
Pb	**0.102 (0.036, 0.168)**	**0.005**	**0.096 (0.030, 0.162)**	**0.013**
Fe	**−0.461 (−0.746**, **−0.177)**	**0.003**	**−0.471 (−0.772**, **−0.170)**	**0.005**
Ca	0.181 (−0.072, 0.435)	0.209	0.226 (−0.034, 0.485)	0.141
Cu	0.230 (−0.011, 0.471)	0.096	0.261 (0.020, 0.502)	0.070
Zn	0.147 (−0.022, 0.316)	0.126	0.175 (0.002, 0.349)	0.090
Mg	−0.263 (−0.543, 0.017)	0.099	−0.187 (−0.481, 0.108)	0.171
**TT3**				
Pb	−0.003 (−0.018, 0.012)	0.670	0.000 (−0.015, 0.015)	0.984
Fe	**0.172 (0.108, 0.236)**	**< 0.001**	**0.163 (0.095, 0.230)**	**< 0.001**
Ca	0.012 (−0.045, 0.069)	0.670	0.004 (−0.055, 0.062)	0.933
Cu	0.056 (0.002, 0.111)	0.072	0.047 (−0.007, 0.101)	0.141
Zn	**−0.065 (−0.103**, **−0.027)**	**0.003**	**−0.083 (−0.122**, **−0.044)**	**< 0.001**
Mg	**0.104 (0.041, 0.168)**	**0.003**	**0.079 (0.013, 0.146)**	**0.044**
**TT4**				
Pb	−0.560 (−1.304, 0.185)	0.192	−0.287 (−1.030, 0.004)	0.518
Fe	**13.712 (10.508, 16.915)**	**< 0.001**	**12.255 (8.872, 0.145)**	**< 0.001**
Ca	1.212 (−1.643, 4.067)	0.450	−0.490 (−3.408, 0.020)	0.795
Cu	**8.973 (6.259, 11.687)**	**< 0.001**	**8.403 (5.694, 0.103)**	**< 0.001**
Zn	−0.920 (−2.825, 0.985)	0.430	−2.096 (−4.045, 0.000)	0.070
Mg	**8.673 (5.518, 11.829)**	**< 0.001**	**5.467 (2.154, 0.077)**	**0.003**
**FT3**				
Pb	0.015 (−0.017, 0.047)	0.437	0.025 (−0.007, 0.057)	0.180
Fe	**0.624 (0.487, 0.761)**	**< 0.001**	**0.615 (0.470, 0.760)**	**< 0.001**
Ca	0.135 (0.013, 0.257)	0.056	0.080 (−0.045, 0.205)	0.268
Cu	0.120 (0.004, 0.236)	0.071	0.098 (−0.018, 0.214)	0.149
Zn	**−0.110 (−0.191**, **−0.028)**	**0.017**	**−0.169 (−0.253**, **−0.086)**	**< 0.001**
Mg	**0.231 (0.096, 0.366)**	**0.003**	0.099 (−0.043, 0.241)	0.233
**FT4**				
Pb	**0.251 (0.136, 0.367)**	**< 0.001**	**0.227 (0.112, 0.343)**	**< 0.001**
Fe	**−1.903 (−2.401**, **−1.405)**	**< 0.001**	**−1.938 (−2.465**, **−1.412)**	**< 0.001**
Ca	**−0.606 (−1.049**, **−0.163)**	**0.016**	−0.449 (−0.903,0.006)	0.094
Cu	−0.169 (−0.591, 0.253)	0.464	−0.092 (−0.514, 0.329)	0.742
Zn	**0.341 (0.045, 0.637)**	**0.048**	**0.494 (0.190, 0.797)**	**0.003**
Mg	−0.213 (−0.704, 0.277)	0.450	0.287 (−0.228, 0.803)	0.330

Metal and thyroid hormone concentrations were ln-transformed. Each model was adjusted for age, gender, BMI, parent smoking, parent history of thyroid disease, maternal education level, urinary iodine concentration and season. The results in bold are statistically significant.

The RCS models showed significant non-linear associations for Fe with TT4 and FT4, Cu with TT4 and FT4 (all *P* for non-linear < 0.05, Supplementary Figure S3). The association between Cu and FT4 showed an inverse U-shape (Supplementary Figure S3).

### 3.3 Association between metal mixture exposure and serum thyroid hormones analyzed by QGC model

**Figure 1:**
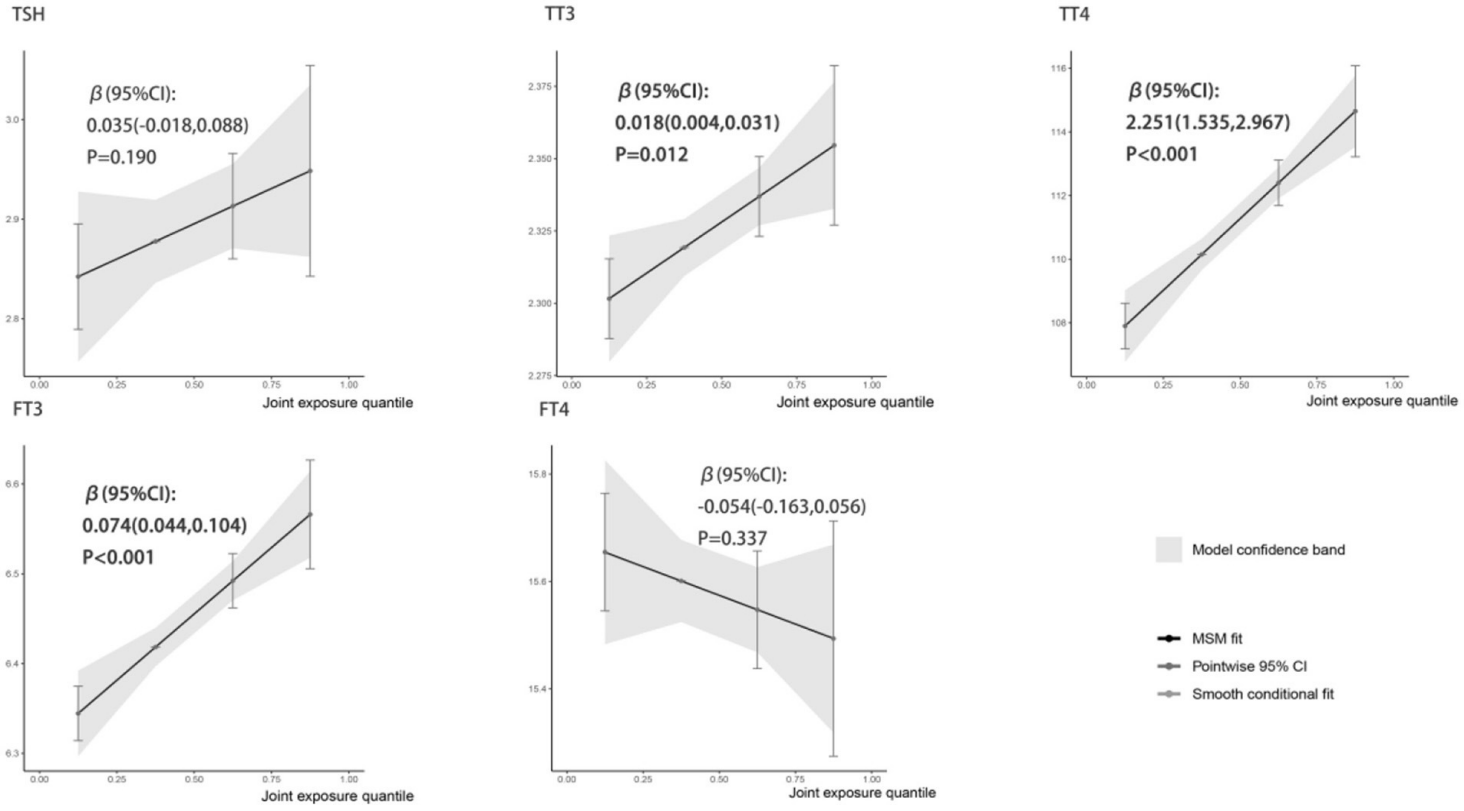
Linear association of the metal mixture with thyroid hormones analyzed by QGC regression. All metal levels were ln-transformed and the models were adjusted by age, gender, BMI, parent smoking, parent history of thyroid disease, maternal education level, urinary iodine concentration and season. Boot = 500: the models with 500 times bootstraps and the results were presented as β (95% CIs).

**Figure 2:**
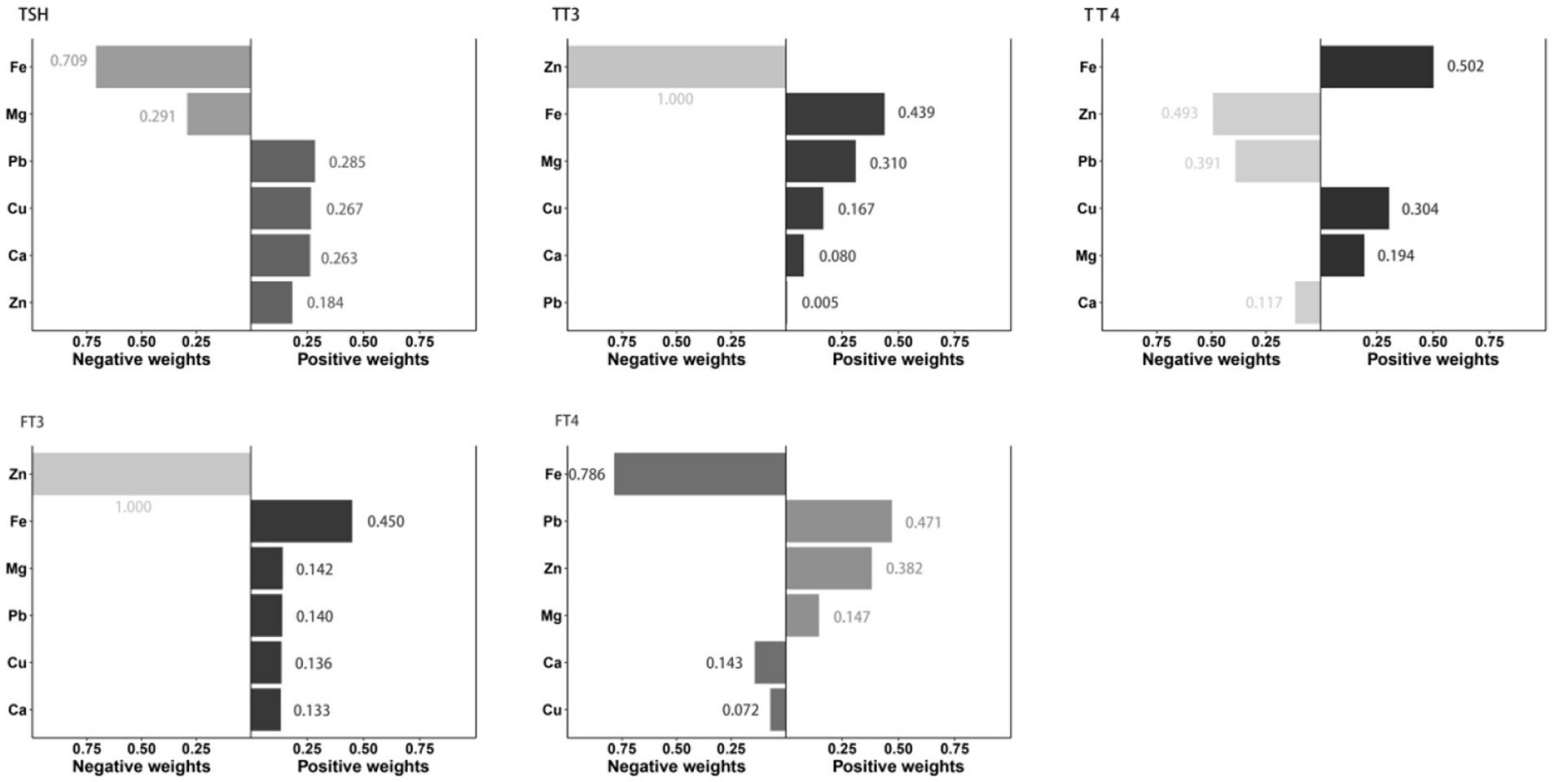
Estimation of individual weight for metal in the mixture associated with thyroid hormone levels. All metal levels were ln-transformed and the models were adjusted by age, gender, BMI, parent smoking, parent history of thyroid disease, maternal education level, urinary iodine concentration and season.

Figure 1 showed the associations between combined six metals exposures and serum thyroid hormones for each higher quartile compared to the lowest quartile. After adjusting the relevant covariates, one quartile increase in selected metal mixtures generated a 0.018-SD increase in TT3 (95% CI: 0.004, 0.031), a 2.251-SD increase in TT4 (95% CI: 1.535, 2.967), and a 0.074-SD increase in FT3 (95% CI: 0.044, 0.104). In Figure 2, the estimated weights of metals for each QGC index in positive and negative direction were indicated, respectively. Fe was the most important positively weighted metal in TT3 (*w* = 0.439), TT4 (*w* = 0.502) and FT3 (*w* = 0.450). Zn was the most important negatively weighted metal in TT3 (*w* = 1.000), TT4 (*w* = 0.493) and FT3 (*w* = 1.000). Fe was both the largest weighted factor for TSH and FT4 (*w* = 0.709 or 0.786).

Supplementary Table S1 presented the association of the 5 essential metal mixture (Fe, Ca, Cu, Zn, and Mg) on thyroid hormones and the weights for each metal in QGC models. One quartile increase in selected metal mixtures generated a 0.019-SD increase in TT3 (95% CI: 0.007, 0.032), a 2.459-SD increase in TT4 (95% CI: 1.848, 3.070), a 0.058-SD increase in FT3 (95% CI: 0.032, 0.084), and a 0.158-SD decrease in FT4 (95% CI: −0.253, −0.062). Fe was the most important positively weighted metal in TT3 (*w* = 0.402), TT4 (*w* = 0.489) and FT3 (*w* = 0.524).

### 3.4 Sensitivity analysis

The results of WQS analyses indicated that concentrations of metal mixture were positively associated with serum TT3 (β = 0.014; *P* < 0.001), TT4 (β = 0.171; *P* < 0.001) and FT3 (β = 0.045; *P* < 0.001) with Fe contributing most to the overall mixture effect (Supplementary Figure S4), which was consistent with the results of QGC regression. Additionally, the concentration of metal mixtures has a significant impact on serum FT4 in both positive (β = 0.064; *P* = 0.002) and negative (β = −0.080; *P* < 0.001) directions. The results of subgroup analyses were similar to the main results and we found no gender-specific and season-specific association in this study (Supplementary Tables S2, S3).

## 4 Discussion

In this cross-sectional study, we explored both individual and joint associations of the selected metallic elements with thyroid hormone profiles in 12,470 children aged 0–14 years. We found that several selected blood metals (Pb, Fe, Cu, Zn and Mg) were associated with changes in one or more parameters of thyroid hormones in the GLR models. Furthermore, we used QGC models to estimate associations of metal mixture and thyroid hormones. Significant positive associations of the metal mixture with TT3, TT4 and FT3 were observed and Fe was identified as the most important contributors, which remained robust in WQS model and stratified by gender and season.

Fe is an essential metallic element that plays critical roles in multiple physiological processes. Our results suggested that Fe was the most influential metallic element for thyroid hormone homeostasis, being positively correlated with TT3, TT4 and FT3 while negatively correlated with TSH and FT4. Similarly, a study conducted in Iran reported that iron supplementation increased TT3 and TT4 levels in iron-deficient non-anemic children (39). A randomized controlled trial in goitrous children showed that Fe-treatment could significantly decrease thyroid size (40). Besides, results among pregnant women showed that blood Fe level was associated with significant increases in FT3 and FT4, as well as decrease in TSH (41). In an animal study, when pregnant rat dams were subjected to Fe deficient from early gestation through weaning, the serum TT3 and TT4 and whole-brain T3 decreased in pups (42). Mechanistic studies shown that Fe could mediate the synthesis of thyroid hormone and conversion of T4 to T3 through increasing the activities of thyroid peroxidase and T4-deiodinase, respectively (43).

Zn probably acts as an essential element involved in the metabolism of thyroid hormones. Research evidence showed that carboxypeptidase, a zinc-dependent enzyme, participated in the synthesis of thyrotropin-releasing hormone (TRH) (44). In children with hypozincemia, Zn sulfate supplementation could improve thyroid function, manifested as decreased TSH level and increased triiodothyronine level (25, 45). Consistently, results of the present study found a positive association between blood Zn and FT4 level. However, there were other investigation with adverse results both among adults and children (26, 46). The present research have not reached a conclusive result yet, probably attribute to the differences in physiological condition and genetic background of participants as well as statistical power and research design.

In this study, blood metals of Pb, Cu and Mg were positively associated with one or more parameters of thyroid hormones including TSH, TT3, TT4 and FT4. In a study of 54 children aged 5–16 years in Slovakia, blood and hair levels of Pb were positively associated with thyroid TSH concentration while negatively associated with FT4 level in the low exposure period (27). Besides, results from children with congenital hypothyroidism in Germany showed that serum Cu levels were associated with significant increases in T3 and T4 levels (47). However, there were also other inconsistent reports (48, 49).

Previous evidence indicated that the combined effect of a multi-metal exposure might differ from the individual effect of single metal, which was more closer to the real exposure scene of human (50). Recently, a cross-sectional investigation among 1,067 adults found no association between metal mixture (Zn, Fe, Ca, Cu, Mg, and Mn) and thyroid function biomarkers (51). Conversely, Gustin et al. reported a positive relationship of joint exposure to iodine, selenium and Zn with TSH, with Zn being the most significant contributor among participants (52). In addition, result of 2007–2012 National Health and Nutrition Examination Survey (NHANES) survey showed that the combined effect of 12 metals was sex-specifically associated with T3, T4 and T3:T4 ratio in adults (53). The present investigation indicated that the joint increases in blood metal concentrations were associated with increased levels of TT3, TT4, and FT3 among children, adding new evidence to the joint effect of metal mixture exposure on thyroid hormone homeostasis. Notably, the results of this study found both significantly positive and negative correlation between metal mixture and FT4 in the WQS regression analysis, which was presumably attributed to complex interactions between elements. Investigation with more refined design and multidisciplinary collaboration are warranted to elucidate the influence of element co-exposure on thyroid hormone level.

Our study has several strengths. Firstly, the relatively large sample size ensures enough statistical power to detect subtle difference. Secondly, to our knowledge, this is the first study to examine the association between multiple metals co-exposures and thyroid hormones in children and identified the most prominent contributor. The result is robust to the adjustment for several covariates and stratification by gender and season. However, our study also has several limitations. Due to the cross-sectional nature, the causal relationships of the blood metal mixture with thyroid hormones remain unclear. Only six metallic elements were included in the model and the potential influence of diabetes status on thyroid hormones was not estimated in this study.

## 5 Conclusion

This study indicated that metal mixture exposure was significantly associated with increased levels of three thyroid function biomarkers (TT3, TT4, and FT3), and Fe was the main contributors among a total of 12,470 Chinese children, which provided evidence for the development of health promotion strategy. However, further prospective cohort and mechanism studies are needed to warrant the conclusions.

## Data Availability

The original contributions presented in the study are included in the article/Supplementary material, further inquiries can be directed to the corresponding authors.
