# Supplementary material for: Associations of combined exposure to selected metal mixtures with thyroid hormones in children: a cross-sectional study in China
Source: Front Public Health. 2025 Jan 22;13:1387702. doi: 10.3389/fpubh.2025.1387702 (PMC11794202; doi:10.3389/fpubh.2025.1387702)
Supplement: Supplementary file 1 [file Data_Sheet_1.pdf]

Supplemental Materials

Associations of exposure to metal mixture with thyroid hormones in children: A cross-sectional study in China

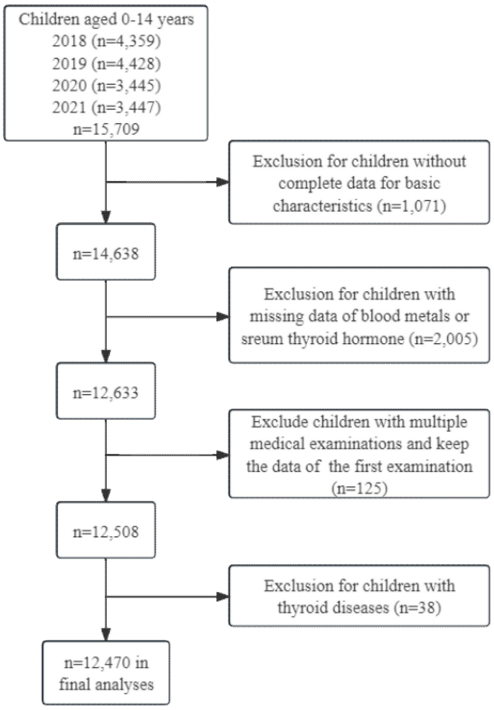

Figure S1. Flowchart for the recruiting study population.

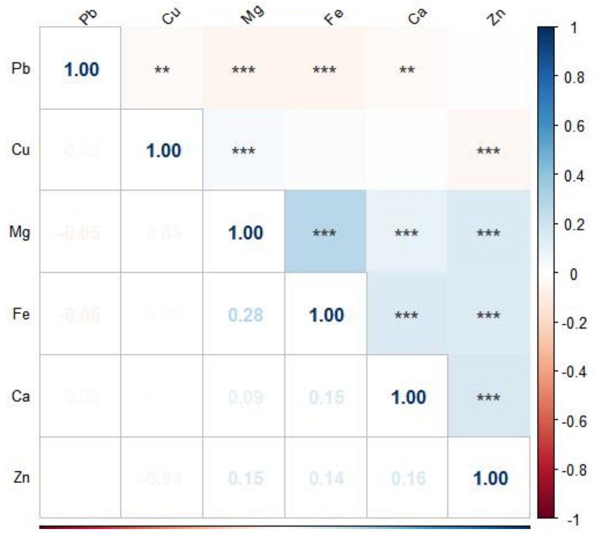

Figure S2. Spearman's correlation analysis among six metals (n = 12,407).

$^{*}P < 0.05$; $^{**}P < 0.01$; $^{***}P < 0.001$

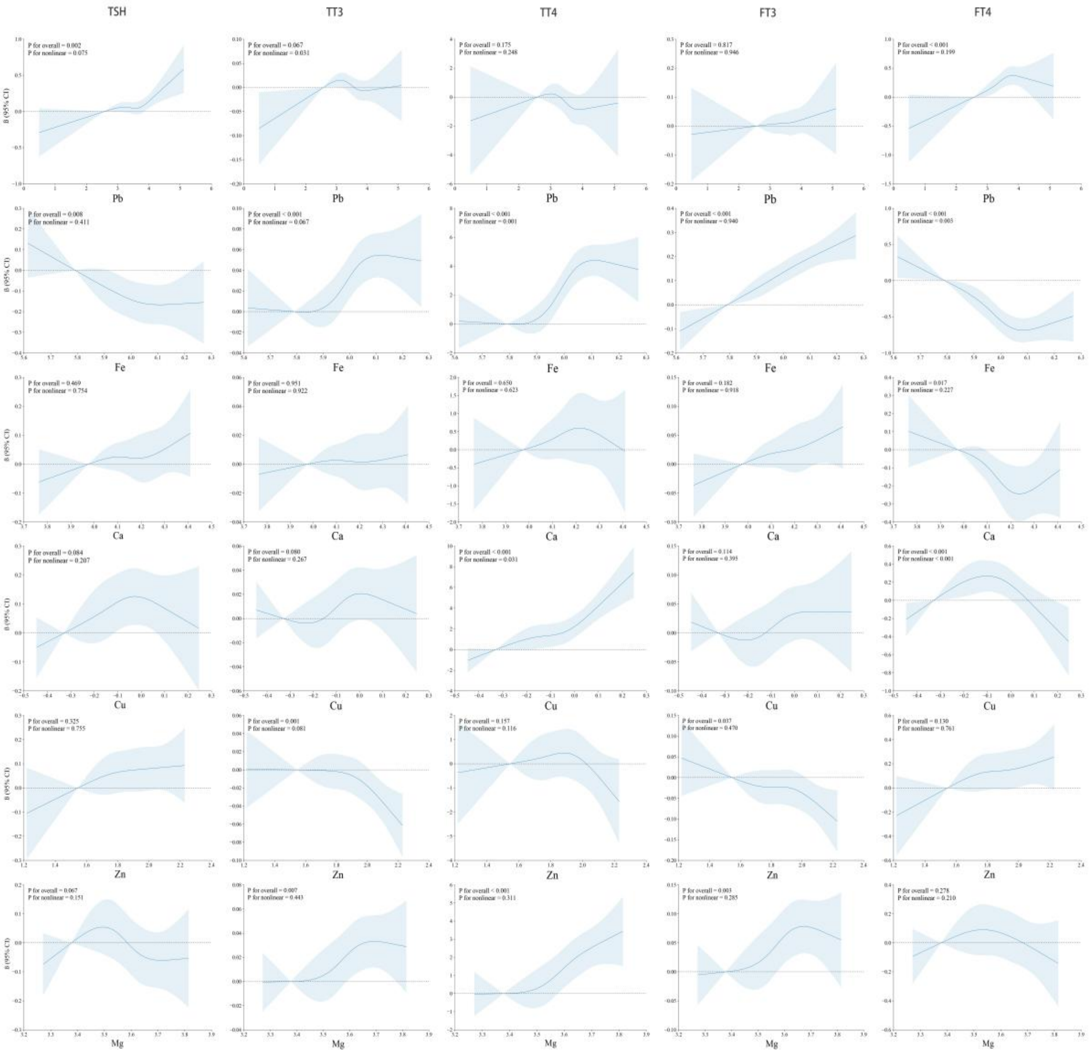

Figure S3. Results of dose-response relationships between metal concentrations and thyroid hormone levels analyzed by restricted cubic spline (n = 12,470).

Table S1 Weights of each essential metal and effect estimate for the essential metal mixture for thyroid hormones analyzed by QGC model.

Metals	TSH	TT3	TT4	FT3	FT4
Fe	-0.720	0.402	0.489	0.524	-0.780
Ca	0.387	0.077	-0.261	0.156	-0.152
Cu	0.345	0.223	0.306	0.159	-0.068
Zn	0.268	-1.000	-0.739	-1.000	0.752
Mg	-0.280	0.298	0.205	0.161	0.248
Metal mixture β (95% CI)	-0.003 (-0.051, 0.045)	**0.019 (0.007, 0.032)***	**2.459 (1.848, 3.070)****	**0.058 (0.032, 0.084)****	**-0.158 (-0.253, -0.062)***

Notes: All metal concentratoins were ln-transformed. Each model was adjusted by age, gender, BMI and season. $^{*}P < 0.05$; $^{**}P < 0.001$.

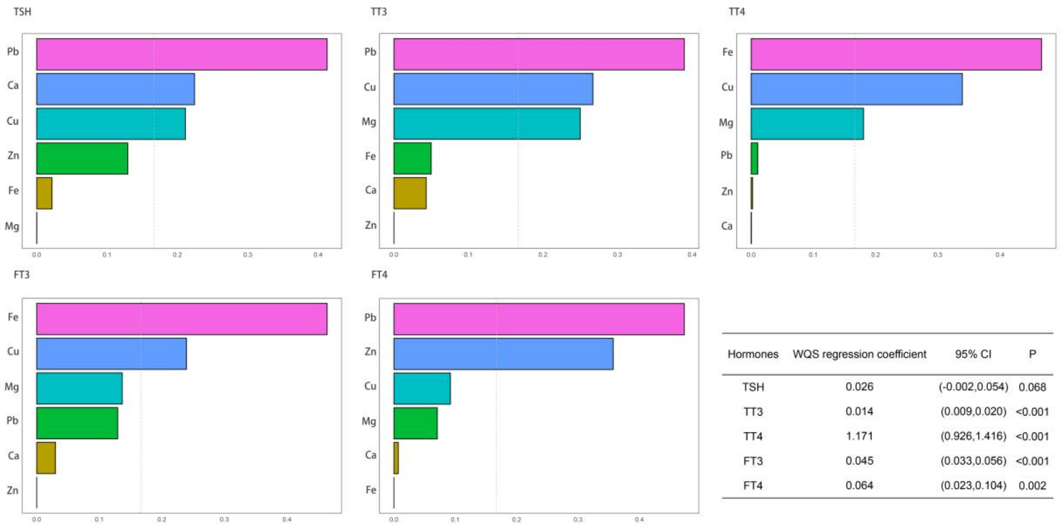

Hormones	WQS regression coefficient	95% CI	P
TSH	0.026	(-0.002,0.054)	0.068
TT3	0.014	(0.009,0.020)	<0.001
TT4	1.171	(0.926,1.416)	<0.001
FT3	0.045	(0.033,0.056)	<0.001
FT4	0.064	(0.023,0.104)	0.002

Figure S4. WQS regression models with positive weights of the metals for thyroid hormones. The models were adjusted by age, gender, BMI, parent smoking, parent history of thyroid disease, maternal education level, urinary iodine concentration and season.

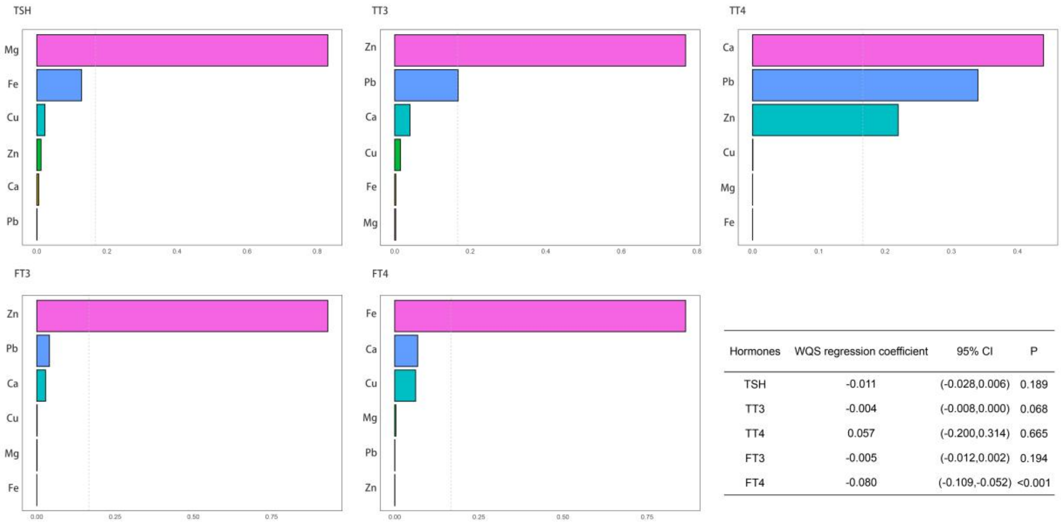

Hormones	WQS regression coefficient	95% CI	P
TSH	-0.011	(-0.028,0.006)	0.189
TT3	-0.004	(-0.008,0.000)	0.068
TT4	0.057	(-0.200,0.314)	0.665
FT3	-0.005	(-0.012,0.002)	0.194
FT4	-0.080	(-0.109,-0.052)	<0.001

Figure S5. WQS regression models with negative weights of the metals for thyroid hormones. The models were adjusted by age, gender, BMI, parent smoking, parent history of thyroid disease, maternal education level, urinary iodine concentration and season.

Table S2. Adjusted regression coefficients and 95% confidence intervals (95% CI) for thyroid hormones with ln-transformed blood metal levels in multiple-metal generalized linear models,

stratified by gender (n = 12,470).

	TSH	TT3	TT4	FT3	FT4
	β (95% CI)	*β* (95% CI)	*β* (95% CI)	*β* (95% CI)	*β* (95% CI)
Pb					
Boy	**0.116(0.031,0.200)***	0.000(-0.019,0.019)	-0.268(-1.235,0.700)	0.035(-0.006,0.075)	**0.208(0.060,0.356)***
Girl	0.071(-0.035,0.177)	0.001(-0.022,0.025)	-0.260(-1.416,0.896)	0.010(-0.041,0.061)	**0.259(0.074,0.445)***
Fe					
Boy	-0.426(-0.807,-0.044)	**0.151(0.065,0.238)***	**13.009(8.641,17.377)****	**0.474(0.291,0.657)****	**-1.655(-2.322,-0.987)****
Girl	-0.486(-0.976,0.004)	**0.186(0.078,0.295)***	**11.074(5.734,16.415)****	**0.863(0.626,0.101)****	**-2.388(-3.245,-1.532)****
Ca					
Boy	0.316(0.002,0.629)	0.021(-0.051,0.092)	-1.418(-5.006,2.169)	0.147(-0.003,0.297)	**-0.749(-1.298,-0.201)***
Girl	0.016(-0.446,0.478)	-0.034(-0.136,0.069)	1.475(-3.563,6.514)	-0.064(-0.288,0.160)	0.162(-0.646,0.970)
Cu					
Boy	0.176(-0.131,0.483)	0.079(0.009,0.149)	**10.647(7.126,14.167)****	0.113(-0.034,0.260)	-0.305(-0.843,0.233)
Girl	0.374(-0.013,0.762)	-0.005(-0.091,0.081)	4.717(0.489,8.945)	0.076(-0.112,0.264)	0.238(-0.440,0.916)
Zn					
Boy	0.152(-0.067,0.370)	**-0.073(-0.123,-0.023)***	-1.633(-4.137,0.872)	**-1.152(-0.257,-0.047)***	0.370(-0.013,0.752)
Girl	0.192(-0.092,0.477)	**-0.104(-0.167,-0.040)***	-3.092(-6.195,0.011)	**-0.198(-0.336,-0.060)***	**0.682(0.185,1.180)***
Mg					
Boy	-0.040(-0.413,0.332)	**0.119(0.034,0.203)***	**5.599(1.333,9.864)***	0.160(-0.018,0.338)	0.177(-0.475,0.829)
Girl	-0.377(-0.860,0.105)	0.018(-0.089,0.125)	5.308(0.048,10.567)	-0.000(-0.234,0.234)	0.486(-0.358,1.329)

Notes: All metal and thyroid hormone levels were ln-transformed. Each model was were adjusted by age, gender, BMI, parent smoking, parent history of thyroid disease, maternal education level, urinary iodine concentration and season. $^{*}P_{FDR} < 0.05$; $^{**}P_{FDR} < 0.001$

Table S3. Adjusted regression coefficients and 95% confidence intervals (95% CI) for thyroid hormones with ln-transformed blood metal levels in multiple-metal generalized linear models, stratified by season (n = 12,470).

	TSH	TT3	TT4	FT3	FT4
	β (95% CI)	*β* (95% CI)	*β* (95% CI)	*β* (95% CI)	β (95% CI)
Pb					
Spring	-0.025(-0.149,0.099)	0.035(0.004,0.066)	-0.401(-1.917,1.114)	-0.016(-0.085,0.054)	**0.534(0.292,0.776)****
Summer	**0.208(0.107,0.309)****	-0.024(-0.046,-0.001)	-1.106(-2.232,0.020)	**0.062(0.015,0.109)***	0.096(-0.082,0.274)
Fall	0.095(0.-031,0.221)	0.004(-0.029,0.038)	1.819(0.088,3.549)	-0.039(-0.107,0.028)	0.241(0.005,0.476)
Winter	-0.056(-0.272,0.159)	0.020(-0.020,0.059)	-0.373(-2.383,1.636)	0.081(-0.007,0.168)	0.165(-0.162,0.491)
Fe					
Spring	-0.196(-0.711,0.319)	**0.415(0.284,0.545)****	**19.648(13.341,25.956)****	**1.141(0.850,1.431)****	**-1.614(-2.621,-0.606)***
Summer	**-0.973(-1.457,-0.489)****	-0.033(-0.142,0.076)	**7.203(1.795,12.611)***	**0.314(0.087,0.542)***	**-2.371(-3.224,-1.518)****
Fall	-0.366(-0.995,0.263)	0.133(-0.034,0.301)	7.537(-1.103,16.178)	0.090(-0.247,0.427)	-1.171(-2.346,0.003)
Winter	-0.048(-0.933,0.837)	**0.251(0.090,0.413)***	**16.294(8.042,24.546)****	**0.896(0.538,1.254)****	**-1.938(-3.279,-0.597)***
Ca					

Spring	0.257(-0.242,0.757)	-0.006(-0.132,0.120)	-3.839(-9.957,2.280)	-0.029(-0.312,0.0.253)	-1.099(-2.076,-0.121)
Summer	0.305(-0.049,0.660)	0.021(-0.059,0.100)	3.172(-0.782,7.126)	0.180(0.014,0.347)	**-0.872(-1.495,-0.248)***
Fall	0.364(-0.254,0.983)	-0.103(-0.268,0.061)	-8.880(-17.378,-0.381)	0.115(-0.216,0.446)	0.917(-0.238,2.071)
Winter	-0.204(-1.102,0.695)	0.084(-0.080,0.248)	-0.920(-9.297,7.458)	-0.123(-0.487,0.240)	0.953(-0.408,2.315)
Cu					
Spring	0.426(0.022,0.831)	0.026(-0.076,0.129)	**7.820(2.866,12.775)***	0.251(0.022,0.479)	-0.145(-0.937,0.646)
Summer	0.146(-0.237,0.529)	**0.110(0.024,0.196)***	**10.172(5.898,14.445)****	0.004(-0.176,0.184)	0.254(-0.420,0.928)
Fall	0.165(-0.347,0.677)	0.017(-0.119,0.153	6.462(-0.576,13.500)	0.031(-0.244,0.305)	-0.893(-1.849,0.063)
Winter	0.304(-0.422,1.030)	-0.043(-0.175,0.090)	7.152(0.381,13.923)	0.130(-0.164,0.424)	-0.145(-1.245,0.956)
Zn					
Spring	0.314(0.008,0.620)	**-0.128(-0.205,-0.050)***	-3.793(-7.542,-0.044)	**-0.284(-0.457,-0.111)***	**0.900(0.301,1.498)***
Summer	0.166(-0.106,0.437)	-0.053(-0.114,0.008)	-2.835(-5.866,0.196)	-0.103(-0.230,0.025)	0.263(-0.215,0.741)
Fall	0.151(-0.220,0.523)	**-0.173(-0.272,-0.074)***	-4.295(-9.405,0.815)	-0.150(-0.349,0.050)	-0.263(-0.957,0.431)
Winter	-0.002(-0.505,0.500)	-0.020(-0.112,0.071)	2.794(-1.888,7.476)	-0.129(-0.332,0.075)	0.896(0.136,1.657)
Mg					
Spring	-0.010(-0.508,0.489)	0.062(-0.064,0.188)	4.364(-1.738,10.465)	-0.133(-0.414,0.148)	0.050(-0.925,1.025)
Summer	-0.124(-0.611,0.364)	0.065(-0.045,0.175)	**7.579(2.133,13.024)***	0.170(-0.059,0.399)	0.392(-0.467,1.251)
Fall	0.097(-0.493,0.688)	0.079(-0.078,0.236)	2.144(-5.969,10.257)	0.200(-0.116,0.517)	0.288(-0.814,1.391)
Winter	-0.597(-1.463,0.270)	0.143(-0.015,0.301)	6.756(-1.322,14.835)	0.268(-0.083,0.618)	0.494(-0.819,1.806)

Notes: All metal and thyroid hormone levels were ln-transformed. Each model was adjusted by age, gender, BMI, parent smoking, parent history of thyroid disease, maternal education level, urinary iodine concentration and season. $^{*}P_{FDR} < 0.05$; $^{**}P_{FDR} < 0.001$.
